# Plume Activity and Tidal Deformation on Enceladus Influenced by Faults and Variable Ice Shell Thickness

**DOI:** 10.1089/ast.2016.1629

**Published:** 2017-09-01

**Authors:** Marie Běhounková, Ondřej Souček, Jaroslav Hron, Ondřej Čadek

**Affiliations:** ^1^ Charles University, Faculty of Mathematics and Physics, Department of Geophysics, Prague, Czech Republic.; ^2^ Mathematical Institute of Charles University, Prague, Czech Republic.

## Abstract

We investigated the effect of variations in ice shell thickness and of the tiger stripe fractures crossing Enceladus' south polar terrain on the moon's tidal deformation by performing finite element calculations in three-dimensional geometry. The combination of thinning in the polar region and the presence of faults has a synergistic effect that leads to an increase of both the displacement and stress in the south polar terrain by an order of magnitude compared to that of the traditional model with a uniform shell thickness and without faults. Assuming a simplified conductive heat transfer and neglecting the heat sources below the ice shell, we computed the global heat budget of the ice shell. For the inelastic properties of the shell described by a Maxwell viscoelastic model, we show that unrealistically low average viscosity of the order of 10^13^ Pa s is necessary for preserving the volume of the ocean, suggesting the important role of the heat sources in the deep interior. Similarly, low viscosity is required to predict the observed delay of the plume activity, which hints at other delaying mechanisms than just the viscoelasticity of the ice shell. The presence of faults results in large spatial and temporal heterogeneity of geysering activity compared to the traditional models without faults. Our model contributes to understanding the physical mechanisms that control the fault activity, and it provides potentially useful information for future missions that will sample the plume for evidence of life. Key Words: Enceladus—Tidal deformation—Faults—Variable ice shell thickness—Tidal heating—Plume activity and timing. Astrobiology 17, 941–954.

## 1. Introduction

Recent years have seen remarkable advances in our understanding of Enceladus' internal structure and south polar jet activity. The presence of an internal liquid water reservoir, suggested by the discovery of jets of water vapor and icy particles by the Cassini spacecraft in 2005 (Porco *et al.,* 2006), has been supported by the analysis of icy grains emanating from Enceladus' south polar faults (Postberg *et al.,* 2009, 2011) and independently supported by the measurements of physical libration (Thomas *et al.,* 2016). The presence of such a deep ocean in contact with a silicate core makes Enceladus one of the most promising habitable spots beyond Earth. The jets emanating from the moon's interior provide a means with which to investigate the conditions in the ocean that can be compared to those that support life as we know it (*e.g.,* Waite *et al.,* 2017).

The thickness of the ice shell and the extent of the ocean have long been a matter of debate. The observed libration amplitude suggests that the ocean is global and the ice shell above the ocean is on average 14–26 km thick (Thomas *et al.,* 2016; van Hoolst *et al.,* 2016), much less than previously expected (Schubert *et al.,* 2007; Iess *et al.,* 2014; McKinnon, 2015). The measurements of Enceladus' topography (Thomas *et al.,* 2007; Nimmo *et al.,* 2011) and low-degree gravity (Iess *et al.,* 2014) indicate that the ice shell is significantly thinned in the southern hemisphere (Iess *et al.,* 2014; McKinnon, 2015) and may be only a few kilometers thick at the south pole (Čadek *et al.,* 2016). These findings have reopened the question as to the nature of the heat sources that maintain the subsurface ocean, with tidal heating remaining the most plausible candidate for a significant heat source. Numerical models concentrate mainly on the heat production within the ice shell. Nevertheless, they suggest that it is difficult to counterbalance the heat loss through the surface of the ocean by the heat produced by tides in the ice shell (*e.g.,* O'Neill and Nimmo, 2010; Běhounková *et al.,* 2012; Shoji *et al.,* 2014) unless heat also generated in the deep interior of Enceladus is considered (*i.e.,* in the ocean and/or in the silicate core) (Tyler, 2009; Roberts, 2015; Travis and Schubert, 2015; Choblet *et al.,* 2016). The existence of such deep sources is further supported by a large dissipation in Saturn, as recently estimated by Lainey *et al.* (2012, 2017), together with the equilibrium (Meyer and Wisdom, 2007) or dynamical (Fuller *et al.,* 2016) models of tides that suggest the equilibrium heat production of up to ∼50 GW.

Considerable progress has also been made in studying the south polar terrain (SPT). With its jets and faults, which are very likely connected to the subsurface ocean, this region provides a means by which to probe the interior of the moon. The jet activity is concentrated along four fractures, informally called “tiger stripes,” and varies both spatially and temporally (Porco *et al.,* 2014). The maximum plume activity is observed near apocenter, which indicates that the activity is modulated by tidal deformation (Hedman *et al.,* 2013; Nimmo *et al.,* 2014). The predicted activity for a tidally loaded elastic ice shell is nevertheless 5 h delayed compared to observations. The timing of the plume has been modeled with different approaches (Hurford *et al.,* 2007; Nimmo *et al.,* 2014; Běhounková *et al.,* 2015; Kite and Rubin, 2016).

Besides the geyser activity, the tiger stripes also show elevated surface temperatures (Spencer *et al.,* 2006; Abramov and Spencer, 2009) with the total heat radiated over the SPT reaching ∼15 GW (Howett *et al.,* 2011) with endogenic power of ∼5 GW along the stripes (Spencer *et al.,* 2013). The analysis of silicon-rich, nanometer-sized dust particles in Saturn's E ring (Hsu *et al.,* 2015) suggests ongoing hydrothermal reactions in Enceladus' core and quick transport of hydrothermal products from the ocean floor up to the plume. This implies that the passage of plume constituents through the ice shell is highly efficient and indicates that the tiger stripes are directly connected to the subsurface ocean.

In the past 10 years, a number of theoretical and numerical models have been developed to quantify the tidal deformation of Enceladus and its tidal heat production (*e.g.,* Tobie *et al.,* 2005, 2008; Meyer and Wisdom, 2007; Wahr *et al.,* 2009) with the intent to address the heat transfer in its ice shell (*e.g.,* Mitri and Showman, 2008a, 2008b; Roberts and Nimmo, 2008; Stegman *et al.,* 2009; Běhounková *et al.,* 2010; Showman *et al.,* 2013; Rozel *et al.,* 2014) and test possible scenarios of its evolution (*e.g.,* O'Neill and Nimmo, 2010; Běhounková *et al.,* 2012; Shoji *et al.,* 2014; Travis and Schubert, 2015). The long-term goal of this research effort is to create a self-consistent model of Enceladus that would explain its current thermal budget and observed geysering activity and provide clues to understanding its geological and thermal history.

In this paper, we focus on the particular problem of the tidal deformation of the ice shell, and for the first time, we present tidal deformation and an estimate of tidal heating for an ice shell of uneven thickness including faults in the SPT. Until recently, this problem has only been solved for a spherical shell of constant thickness and a simplified (mostly radially dependent) distribution of material parameters (*e.g.,* Tobie *et al.,* 2005; Wahr *et al.,* 2009). Only a few studies have investigated the influence of lateral variations in viscosity on the tidal heating pattern (Tobie *et al.,* 2008; Běhounková *et al.,* 2010; Han and Showman, 2010), but these studies did not include the effect of the tiger stripes because of the lack of resolution. Large ice shell thickness variations have recently been suggested by the analysis of gravity, topography (Iess *et al.,* 2014; McKinnon, 2015), and libration data (Beuthe *et al.,* 2016; Čadek *et al.,* 2016; Thomas *et al.,* 2016; van Hoolst *et al.,* 2016), and there is growing evidence that the tiger stripes are actually fissures that pass through the whole thickness of the ice shell (Hsu *et al.,* 2015; Kite and Rubin, 2016). As we will see, both these effects—thickness variations and faults—are important, and they can dramatically influence the distribution of stress in the SPT and possibly the spatial-temporal activity of the plume as well.

The material parameters that control the tidal deformation of the ice shell are elastic moduli and viscosity (*e.g.,* McCarthy and Castillo-Rogez, 2013). The viscosity of ice is a complex function of temperature, stress, and grain size, and it may vary spatially by orders of magnitude (*e.g.,* Goldsby and Kohlstedt, 2001; Duval, 2013). Consequently, the uncertainties in these parameters limit the present-day knowledge of viscosity distribution. The viscosity of ice is likely to decrease to ∼10^14^ Pa s at the base of the ice shell and can be even lower if the ice matrix contains a few percent of meltwater (De La Chapelle *et al.,* 1999) or if the grain size is very small (<1 mm). In the cold, uppermost part of the shell the viscosity of ice is presumably very high (>10^20^ Pa s), but an accurate estimate of inelastic properties of ice valid at tidal timescale is not known.

Compared to viscosity, the elastic moduli of ice only weakly depend on temperature (Proctor, 1966), and they are basically independent of stress and grain size. However, their effective values can significantly decrease in regions that do not transmit stress, such as fractured zones or faults. This property was employed by Souček *et al.* (2016), who incorporated the tiger stripes in terms of narrow zones that pass vertically through the ice shell, in which the elastic moduli were artificially reduced by several orders of magnitude. Souček *et al.* (2016) avoided the use of the standard spectral method, which does not allow for a sufficiently high resolution; instead these authors applied the finite element method on an unstructured grid. In the present study, we followed the same approach but also included the variations of ice shell thickness based on the model of Čadek *et al.* (2016), so we discuss here the joint effect of faults and ice thickness variations on Enceladus' tidal deformation, thermal budget, and plume activity.

The structure of this paper is as follows: In Section 2, we briefly summarize the method by Souček *et al.* (2016) and define four structural models of Enceladus' ice shell. In the sections that follow, we compute the tidal response for these models and discuss the surface distribution of stress and displacement (Section 3), and we estimate the tidal heat produced by these models and compare it with the conductive heat loss through the ice shell (Section 4). We then compare the predicted spatial and temporal jet activity at the tiger stripes with available observations (Section 5). In the final section, we summarize the results and outline the implications of our findings.

## 2. Method and Models

We adopted the numerical method developed by Souček *et al.* (2016), who investigated the tidal deformation of Enceladus' ice shell of uniform thickness including faults in the SPT. Here, we have extended the methodology to include two additional features: variable ice shell thickness and 1:1 physical libration. The implementation of the variable thickness requires the boundary conditions to be adjusted for the irregular geometry of the boundaries. The radial unit vector used in the formulation for a shell of uniform thickness (see the supplementary information to Souček *et al.* [2016]) must be replaced by the unit vector normal to the (generally aspherical) boundary.

The second modification of the numerical method is the correction of the tidal force for the effect of libration. The tidal force that drives Enceladus' deformation originates in its synchronous rotation on a slightly eccentric orbit around Saturn. Neglecting inclination, the longitude evolution of the sub-saturnian point *φ*_S_ is approximately described in the works of, for example, Hurford *et al.* (2009) and Nimmo *et al.* (2014) as follows:

\begin{align*}
{ \varphi _{ \rm{S}}} ( t ) \approx 2e \ { \rm{sin}} ( nt ) - { \gamma _0} \ { \rm{sin}} \left( {nt} \right) \tag{1}
\end{align*}

where *t* is the time, *e* = 0.0045 is the eccentricity of the orbit, *n* = 5.310^−5^ s^−1^ is the mean motion, and *γ*_0_ = 0.12° is the amplitude of 1:1 in phase physical libration due to the gravitational interaction with Saturn (*cf.* Thomas *et al.,* 2016). The expression for the tidal potential including the effect of libration can then be derived with the standard procedure (*e.g.,* Kaula, 1964) in which the time evolution of *φ*_S_ is given by Eq. 1. In all the numerical simulations, we consider only the time-dependent part of the tidal potential at spherical harmonic degree 2. We find that the inclusion of in-phase libration reduces the amplitudes of stress and displacement by about 20% compared to the models without libration but has only a minor effect on the deformation pattern. Note that in Eq. 1 we omit the long-period libration due to the interaction with the neighboring satellites. Since the long-period libration acts on a 5–10 year timescale (Rambaux *et al.,* 2010), its variations during a single orbit are very small and have little impact on the short-period phenomena studied in the paper.

All the calculations of tidal deformation presented in this study are performed assuming elastic behavior of ice. The elastic models provide a reasonable estimate of stresses, but they cannot be directly used to assess the inelastic effects, such as tidal heating and the phase lag of jet activity. These effects are estimated by using the correspondence principle, which gives sufficiently accurate results provided that the ratio of viscosity to rigidity is constant in space (for details, see Souček *et al.,* 2016).

**FIG. 1.**
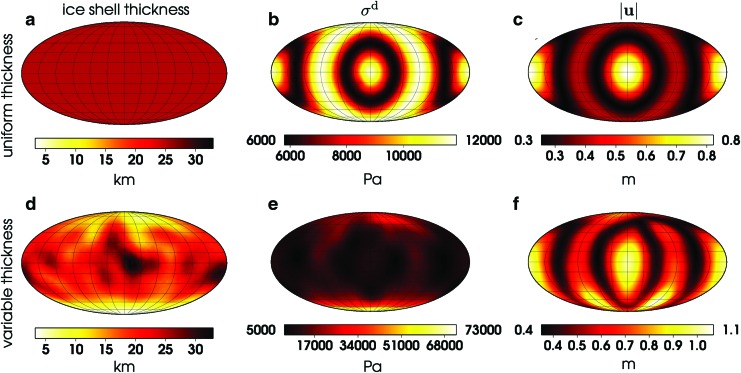
Influence of variable ice shell thickness on the magnitude of deviatoric stress (*σ*^d^) and displacement (|**u**|) at the surface. Panels **a**–**c** correspond to model M^*U*^ (uniform thickness, without faults), while model M^*V*^ (variable thickness, without faults) is illustrated in panels **d**–**f**. Left (a, d), middle (b, e), and right (c, f) panels show the ice shell thickness, the magnitude of deviatoric stress tensor, and the magnitude of displacement, respectively. The stress and displacement maps are computed at periapsis assuming elastic behavior of ice. The maps are shown in the Mollweide projection with the sub-saturnian point at the center.

We examine the individual and joint effects of faults and ice thickness variations on Enceladus' tidal deformation by using four models of the ice shell, of variations in shape (uniform or variable ice thickness), and of internal structure (without or with faults). The first model, hereinafter referred to as M^*U*^, has a uniform thickness of 25 km, and it is without faults. The tidal deformation computed for this model is used for comparison with other, more complex models and allows for quantification of the relative contribution of faults and/or thickness variations to the total deformation of the ice shell. The second model, M^*V*^, does not include faults, but its shell thickness varies in agreement with the model of Čadek *et al.* (2016). The ice shell is 22 km thick on average, but the thickness is reduced around the poles and decreases to only 3 km in the SPT. In equatorial regions, the ice thickness ranges from 20 km to more than 30 km (see also Fig. 1d). The last two models, denoted by M^*UF*^ and M^*VF*^, include faults and have the same domain geometry as M^*U*^ and M^*V*^, respectively. The faults are mimicked by narrow (∼1 km thick) zones that pass vertically through the ice shell, in which the elastic moduli are reduced by 5 orders of magnitude. The geometry of the prescribed weak zones approximately corresponds to the positions of the recently active faults (Porco *et al.,* 2014). As discussed in more detail by Souček *et al.* (2016), the weak zones in our model can be regarded as the limiting case corresponding to geological faults with negligible frictional and bulk resistance.

The applied methodology relies on several simplifying assumptions. First, we apply a quasi-static approximation that solves the time evolution as a sequence of static equilibrium problems. Additionally, we do not consider any variations of material properties with depth, and we neglect the effects of self-gravitation and density anomalies due to volumetric deformation. We also omit the potential impact of fluid motion within the faults (*cf.* Kite and Rubin, 2016), possible variations in the fault geometry with depth, as well as the contact conditions on the faults. For all models considered, the deformation is computed with the open source finite element software package FEniCS (Alnaes *et al.,* 2015). The details of numerical implementation can be found in the supplementary information of Souček *et al.* (2016). The model parameters are listed in Table 1.

**Table 1.** List of Model Parameters

Shear modulus	3.3 · 10^9^ Pa
Poisson ratio	0.33
Ice density	925 kg m^−3^
Water density	1007 kg m^−3^
Orbital period	1.37 days
Eccentricity	0.0045
Radius	252 km
Ice shell thickness	uniform 25 km or variable 3–33 km
Amplitude of short-period libration	0.12°

## 3. Deformation and Stress

In this section, we discuss the impact of ice thickness variations and faults on the displacement and stress field. As illustrated in Fig. 1a–1c, the elastic deformation of the ice shell model M^*U*^ (uniform thickness, without faults) due to tidal loading is perfectly correlated with the quadrupole distribution of the tidal potential. The stress and displacement fields are symmetric about the equator and, at periapsis, also about the prime meridian. The stress does not exceed 12 kPa, and the maximum displacement at the surface is about 0.8 m.

**FIG. 2.**
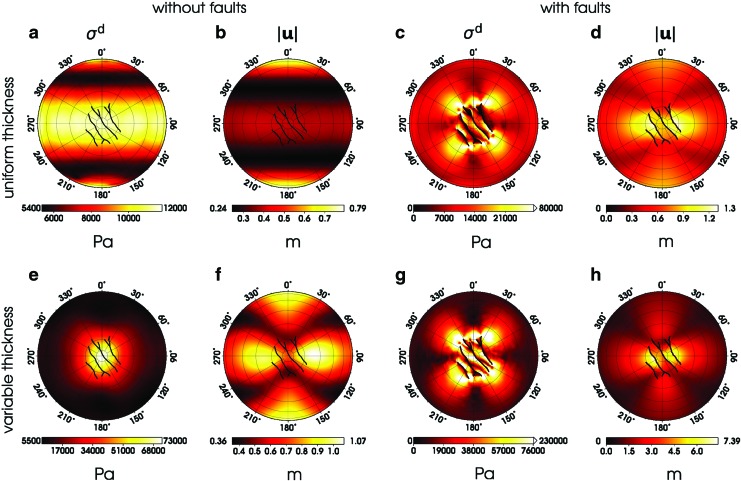
Magnitude of deviatoric stress (*σ*^d^) and displacement (|**u**|) at the surface of the southern hemisphere computed at periapsis for models M^*U*^ (panels **a** and **b**), M^*UF*^ (**c**, **d**), M^*V*^ (**e**, **f**) and M^*VF*^ (**g**, **h**). Positions of the faults (“tiger stripes”) are indicated by the black lines. The faults are plotted also in the maps where the faults were not considered in calculating the stress and displacement field. All maps are shown in the orthographic projection.

The results for the model M^*V*^ (variable thickness, without faults) computed at the same orbital phase are plotted in Fig. 1d–1f. A comparison of these results with those obtained for model M^*U*^ suggests that the variations in the ice shell thickness significantly influence the distribution of stress, while the surface displacement is only weakly affected. The local amplitude of stress is controlled by the local ice shell thickness, and compared to model M^*U*^, it is strongly (by a factor of about 6) increased in the SPT where the ice thickness reaches its minimum. In a first approximation, the magnitude of stress is inversely proportional to the ice thickness, and the stress pattern in Fig. 1e could be estimated from that in Fig. 1b if scaled by the thickness ratio 25/*D,* where *D* is the thickness of ice given in Fig. 1d. Compared to the stress, the increase of displacement around the south pole is mild (by a factor up to 2), and the global pattern of the displacement (Fig. 1f) does not differ much from the pattern obtained for model M^*U*^ (Fig. 1c). The detailed distribution of stress and displacement on the southern hemisphere for models M^*U*^ and M^*V*^ is shown in Fig. 2, panels a, b and e, f, respectively.

The effect of faults on the tidal deformation of an ice shell of constant thickness (model M^*UF*^) is illustrated in panels c and d of Fig. 2. The displacement field is mainly affected in the SPT where the magnitude of displacement increases by a factor of 2–3 compared to model M^*U*^ (for the time evolution of displacement over the whole orbit, see animations in the supplementary information of Souček *et al.* [2016]). The displacement in the northern hemisphere remains basically unaffected by their presence. The transition zones between the broken blocks with enhanced displacement and the compact parts of the ice shell with only moderate displacement therefore suffer from large stress. The stress field in the SPT has a small-scale structure with pronounced (∼80 kPa) maxima around the tips of the faults. Since the faults are designed as stress free in our model, the areas of enhanced stress do not correlate with the locations of the faults, but they are concentrated around the SPT boundary.

The inclusion of both faults and ice shell thickness variations (model M^*VF*^) has a synergistic effect on the deformation in the SPT. The displacement at the faults (Fig. 2h) locally exceeds 7 m, which is about 7 times more than for model M^*V*^ and 5.5 times more than for model M^*UF*^. The stress pattern in the SPT (Fig. 2g) is controlled by the geometry of the faults, and it is thus similar to that obtained for model M^*UF*^. However, the stress amplitudes are significantly larger than in model M^*UF*^, and they exceed 100 kPa in a relatively large area. The joint effect of the faults and the ice shell thickness variations is only strong in the SPT and its vicinity. Outside the SPT region, the stress gradually decreases, and its distribution on the northern hemisphere is similar to that obtained for model M^*V*^ (Fig. 1e).

The stress in the shell predicted by our simulations does not exceed the tensile strength of ice (*e.g.,* Schulson, 2001; Lee *et al.,* 2005), with the possible exception at the tips of the faults, and thus does not seem to provide a mechanism for the fracture initiation. Nevertheless, we take into account only stresses corresponding to the short-period deformation, and we do not include stress variations related with processes on longer timescales, such as volumetric changes due to melting and crystallization (*e.g.,* Běhounková *et al.,* 2012), tectonic processes (*e.g.,* Schenk and McKinnon, 2009; Crow-Willard and Pappalardo, 2015), or variations of rotational parameters (*e.g.,* Nimmo and Pappalardo, 2006; Matsuyama and Nimmo, 2008; Martin, 2016).

To allow for comparison with previous works, we have evaluated the potential Love number *k*. In the case of the model without faults and with uniform ice shell thickness M^*U*^, we obtain *k*_2_ = 0.014 (in agreement with, *e.g.,* Wahr *et al.,* 2009). Due to the aspherical character of other models, we observe splitting of the Love number *k_lm_* defined for degree *l* and order *m* as a ratio of amplitudes of the additional and the forcing potentials at given degree and order, averaged over period. At degree 2, we obtain *k*_20_ = 0.016, *k*_22_ = 0.019 for M^*UF*^; *k*_20_ = 0.017, *k*_22_ = 0.021 for M^*V*^; *k*_20_ = 0.026, *k*_22_ = 0.035 for M^*VF*^. In all cases, the Love number increases compared to the spherically symmetric model M^*U*^, and the increase is more pronounced for order 2 (*k*_22_). We also observe contribution to the gravitational potential on higher degrees. The spectral power at degree 3 is approximately 6% of power at degree 2 for model M^*VF*^, 2% for model M^*UF*^, and less than 1% for model M^*V*^.

## 4. Tidal Heating

Despite the fact that the considered ice shell model is purely elastic, we can use the computed stresses to evaluate the dissipation for a Maxwell viscoelastic body, by invoking the correspondence principle and assuming that the Maxwell time (ratio of viscosity to rigidity) is constant in space (Souček *et al.,* 2016). The volumetric tidal heating outside the faults *Q*(*r, ϑ, φ*) is represented by a surface heat flux equivalent

\begin{align*}
 { q_ { { \rm { tidal } } } } \left( { \vartheta , \varphi } \right) = \frac { 1 }  { { r_ { \rm { s } } ^2 \left( { \vartheta , \varphi } \right) } } \int_ { { r_ { \rm { b } } } \left( { \vartheta , \varphi } \right) } ^ { { r_ { \rm { s } } } \left( { \vartheta , \varphi } \right) } { Q \left( { r , \vartheta , \varphi } \right) { r^2 } { \rm { d } } r } \tag { 2 } 
\end{align*}

\begin{align*}
 { \rm { where } } \ Q = \frac { 1 }  { T } \int_0^T { { \frac { { \sigma ^ { \rm { d } } } : { \sigma ^ { \rm { d } } } }  { 2 \eta } } } { \rm { d } } t
\end{align*}

*r* is the radius, *ϑ* and *φ* are the cartographic coordinates on Enceladus, *T* is the orbital period, *η* represents the shear viscosity, and *r*_s_ and *r*_b_ are the local radii of the surface and the ice/water interface, respectively.

**FIG. 3.**
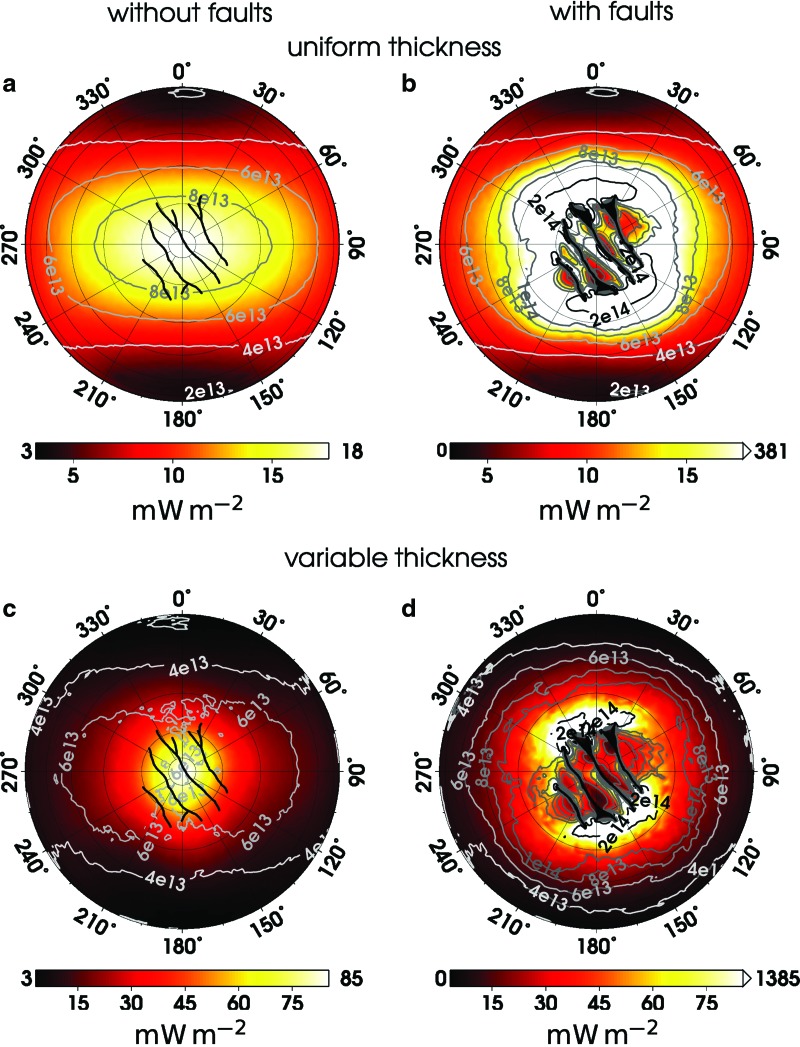
Color maps show tidal heat production, Eq. 2, estimated for viscosity *η* = 10^14^ Pa s and models (**a**) M^*U*^, (**b**) M^*UF*^, (**c**) M^*V*^, and (**d**) M^*VF*^. The gray lines, marking the boundary between melting and freezing regions, are computed for *f* = 1 and different values of viscosity ranging between 10^13^ Pa s and 2 · 10^14^ Pa s.

The tidal heat production computed under this assumption for viscosity of 10^14^ Pa s is shown in Fig. 3. The volumetric tidal heating is proportional to the time average of the square of the deviatoric stress; consequently, the spatial patterns of the tidal heat flux in Fig. 3 are similar to the corresponding distributions of stress in Fig. 2. The heating has a predominantly long-wavelength pattern for models without faults (M^*U*^ and M^*V*^, Fig. 3a and 3c, respectively), with the maximum located on the pole. In model M^*V*^ (Fig. 3c), the heating is further enhanced in the area where the thickness is significantly reduced, approximately corresponding to the SPT. If the faults are included (models M^*UF*^ and M^*VF*^, Fig. 3b and 3d, respectively), the heat flux is focused in the vicinity of the faults' tips and around the boundary of the SPT, and decreases in between the faults. The maximum values of heat flux obtained for viscosity 10^14^ Pa s range from about 20 mW m^−2^ in model M^*U*^ to more than 1000 mW m^−2^ in model M^*VF*^. It should be noted that the values stated above correspond to the viscosity 10^14^ Pa s and therefore scale as $\frac { { { { 10 } ^ { 14 } } { \rm { Pa s } } } }  { \eta }$ if the viscosity value *η* is assumed.

The computed estimates of the tidal heat production inside the ice shell can be used to assess the thermal stability in terms of local conditions at the base of the shell. Since convective heat flow within the thin ice shell is not very likely (Barr and McKinnon, 2007), the thermal state of the shell is expected to be governed by conductive heat transfer and controlled by the distribution of heat sources. Using the analytical solution for the conductive heat transfer (Carslaw and Jaeger, 1959) and our estimates of the tidal heating, we can calculate the 1-D steady-state temperature profile for each point at the surface considering constant heat conductivity (*k* = 2.3 W m^−1^ K^−1^) and the surface and basal temperatures equal to *T*_s_ = 73 K and *T*_b_ = 273 K, respectively. We neglect lateral heat fluxes and assume that the shell is thin [(*r*_s_ − *r*_b_)/*r*_s_ ≫ 1] so that we can solve the heat transfer in Cartesian geometry. Under this simplification, we can express the steady-state basal heat flux as *q*_basal_ = *q* − *q*_tidal_/*f,* where *q*(*ϑ, φ*) = -*k*(*T*_s_ − *T*_b_)/[*r*_s_(*ϑ, φ*) − *r*_b_(*ϑ, φ*)] is the conductive heat flux in the absence of heat sources, and the factor *f* (hereafter referred to as “the heating factor”) characterizes the radial distribution of heat sources. It can be shown that the heating factor *f* ranges between 1 and 2 for heating profiles increasing with depth, a situation characteristic of tidal heating. The heating factor *f* = 1 corresponds to the extreme case when all the heat sources are condensed at the base of the ice shell, while *f* = 2 corresponds to the uniform heat source distribution.

Comparing the basal heat flux *q*_basal_ for a chosen value of viscosity with the heat flux from the deep interior, we can infer the nature of the phase transition at the ice/water interface. Crystallization occurs in regions where the basal heat flux in the ice shell exceeds the flux from the ocean. Conversely, the ice shell is melting if more heat is provided by interior dissipation than required for maintaining the conductive steady-state. Since the heat flux from the ocean is not known, we consider the most extreme case where the heat flux from below is zero (corresponding to zero heating in Enceladus' deep interior). In that case, the equilibrium line between the freezing and melting regions is given by a simple relation *q*_tidal_ = *fq*. Areas where *q*_tidal_ < *fq* are characterized by crystallization at the base of the shell, while areas where *q*_tidal_ > *fq* are prone to basal melting. The exact location of the boundary between the areas depends on viscosity—the lower value of viscosity is considered to indicate the larger area that is affected by melting. Note that basal melting/freezing does not necessarily imply local ice shell thinning/thickening since this process can be compensated dynamically by a lateral flow in the shell (Collins and Goodman, 2007; Čadek *et al.,* 2017; Kamata and Nimmo, 2017).

It is instructive to describe the considered models and their stability in terms of two global thermal characteristics—the power of the energy flow from the ocean *P*_basal_ and the power of tidal dissipation *P*_tidal_. *P*_basal_ is computed as the basal surface integral of *q*_basal_ obtained for the two cases—*f* = 1 (condensed sources) and *f* = 2 (uniform source distribution)—and *P*_tidal_ is the volume integral of the tidal dissipation. The sign of *P*_basal_ determines whether the overall melting exceeds the overall freezing (*P*_basal_ < 0 implies that melting prevails). The values of *P*_basal_ and *P*_tidal_ obtained for different values of ice shell viscosity and the four models considered in this study are summarized in Table 2. If the heating factor *f* = 2 is taken into consideration, the transition from global melting to global freezing occurs between 2 · 10^13^ Pa s and 3 · 10^13^ Pa s corresponding to values of tidal heating ∼30 GW. For the heating factor *f* = 1, the stability region is shifted to the viscosity 4–6 · 10^13^ Pa s and to the tidal heating equal to ∼15 GW. The latter value of heating agrees with the results of Kamata and Nimmo (2017) obtained for radially dependent viscosity. Our results also show that, compared to model M^*U*^, the tidal heating is enhanced by 15–20% if the faults are included and by 40–50% if the reduction of the ice shell thickness in the SPT is taken into account.

**Table 2.** Global Tidal Heating *P*_tidal_ in the Ice Shell and Global Heat Flux at the Base of the Shell *P*_basal_ for Two Extreme Cases: Uniform Volumetric Tidal Heating (*f* = 2) and Heating Condensed at the Base of the Shell (*f* = 1)

	*M*^U^			*M*^V^			*M*^UF^			*M*^VF^		
	P_*tidal*_*GW*	P_*basal*_^f*=1*^*GW*	P_*basal*_^f*=2*^*GW*	P_*tidal*_*GW*	P_*basal*_^f*=1*^*GW*	P_*basal*_^f*=2*^*GW*	P_*tidal*_*GW*	P_*basal*_^f*=1*^*GW*	P_*basal*_^f*=2*^*GW*	P_*tidal*_*GW*	P_*basal*_^f*=1*^*GW*	P_*basal*_^f*=2*^*GW*
1 · 10^13^ Pa s	72.7	−57.8	−21.4	82.9	−68.0	−26.5	102.6	−81.0	−29.7	127.7	−106.1	−42.2
2 · 10^13^ Pa s	36.4	−21.4	−3.2	41.5	−26.5	−5.8	51.3	−29.7	−4.0	63.9	−42.2	−10.3
4 · 10^13^ Pa s	18.2	−3.2	5.9	20.7	−5.8	4.6	25.7	−4.0	8.8	31.9	−10.3	5.7
6 · 10^13^ Pa s	12.1	2.8	8.9	13.8	1.1	8.0	17.1	4.5	13.1	21.3	0.4	11.0
8 · 10^13^ Pa s	9.1	5.9	10.4	10.4	4.6	9.8	12.8	8.8	15.2	16.0	5.7	13.7
1 · 10^14^ Pa s	7.3	7.7	11.3	8.3	6.6	10.8	10.3	11.4	16.5	12.8	8.9	15.3
2 · 10^14^ Pa s	3.6	11.3	13.1	4.1	10.8	12.9	5.1	16.5	19.1	6.4	15.3	18.5
1 · 10^15^ Pa s	0.7	14.2	14.6	0.8	14.1	14.5	1.0	20.6	21.1	1.3	20.4	21.0

The negative and positive sign of *P*_basal_ corresponds to overall melting and crystallization, respectively. Gray cells indicate the global melting regime.

To provide the estimate of minimum tidal heating needed for the stability of the ice shell in the absence of deep heat sources, we restrict the further discussion to the extreme case of *f* = 1. The boundary between melting/crystallization regions is marked by gray lines in Fig. 3 for viscosity between 10^13^ Pa s and 2 · 10^14^ Pa s. In model M^*U*^ (Fig. 3a), heat loss generally exceeds heat production within the shell for viscosities larger than 6 · 10^13^ Pa s, implying that the ocean freezes over the whole area of the ice/ocean boundary. With decreasing viscosity, a melting region forms around the poles and gradually spreads toward the equator. For viscosity 2 · 10^13^ Pa s, the ice shell tends to melt everywhere except for two limited areas at the equator where the tidal heating reaches the minimum. A similar dependence—with minor differences induced by the local thickness—is obtained for the variable thickness model M^*V*^ (Fig. 3c). Although the heat production in the SPT is significantly higher than in model M^*U*^, the viscosity required to reach the melting is somewhat lower (4 · 10^13^ Pa s) than in the previous case because the heat loss in the SPT is larger due to a smaller ice shell thickness. However, as in model M^*U*^, melting occurs over the entire area of the ice/water boundary if viscosity drops to 2 · 10^13^ Pa s.

The melting/freezing pattern becomes more complex if the faults are included (Fig. 3b, 3d). Melting at the tips of the faults that alter with freezing zones in between the fault lines is obtained already for viscosity of ∼2 · 10^14^ Pa s. If the viscosity is only slightly lower (10^14^ Pa s), the tidal heating dominates over the conductive cooling throughout the SPT. Note that our model takes into account neither frictional heating at the faults nor the energy carried by the jets, and it omits the lateral heat flux, which may be large in the vicinity of faults. These effects, if included in the model, would further enhance the melting in the near-fault region.

To summarize, our results indicate that a global thermal equilibrium is possible for conservative values of viscosity (≳ 10^14^ Pa s) only if additional heat sources of the magnitude of tens of gigawatts are present in Enceladus' deep interior. However, none of the thermal models discussed above is stable locally. The local stability would require the heat flux from the ocean to be laterally heterogeneous to balance the heat flux *P*_basal_ at the base of the ice shell, and/or the changes in the ice shell thickness due to melting/crystallization would have to be compensated by lateral mass and heat flow within the shell (Collins and Goodman, 2007; Čadek *et al.,* 2017; Kamata and Nimmo, 2017).

## 5. Activity and Plume Timing

Here, we examine the implications of variable ice shell thickness and inclusion of faults on the activity of the faults. Measurements of the brightness of the plume emanating from the faults provided by the Visible and Infrared Mapping Spectrometer (VIMS; Hedman *et al.,* 2013) and the Imaging Science Subsystem (ISS; Nimmo *et al.,* 2014) report maximum activity near the apoapsis and minimum around periapsis. Traditionally, the activity is interpreted in terms of the normal component of the traction force along the faults—the activity increases in the tensile regime and decreases in the compressional regime (Hurford *et al.,* 2007; Nimmo *et al.,* 2014; Běhounková *et al.,* 2015). The same methodology is applied here for models without the faults (M^*U*^ and M^*V*^). For models where the faults are implemented (M^*UF*^ and M^*VF*^), we can directly evaluate the faults' opening or closure by investigating the jump in the normal displacement along the faults (positive for opening, negative for closing).

**FIG. 4.**
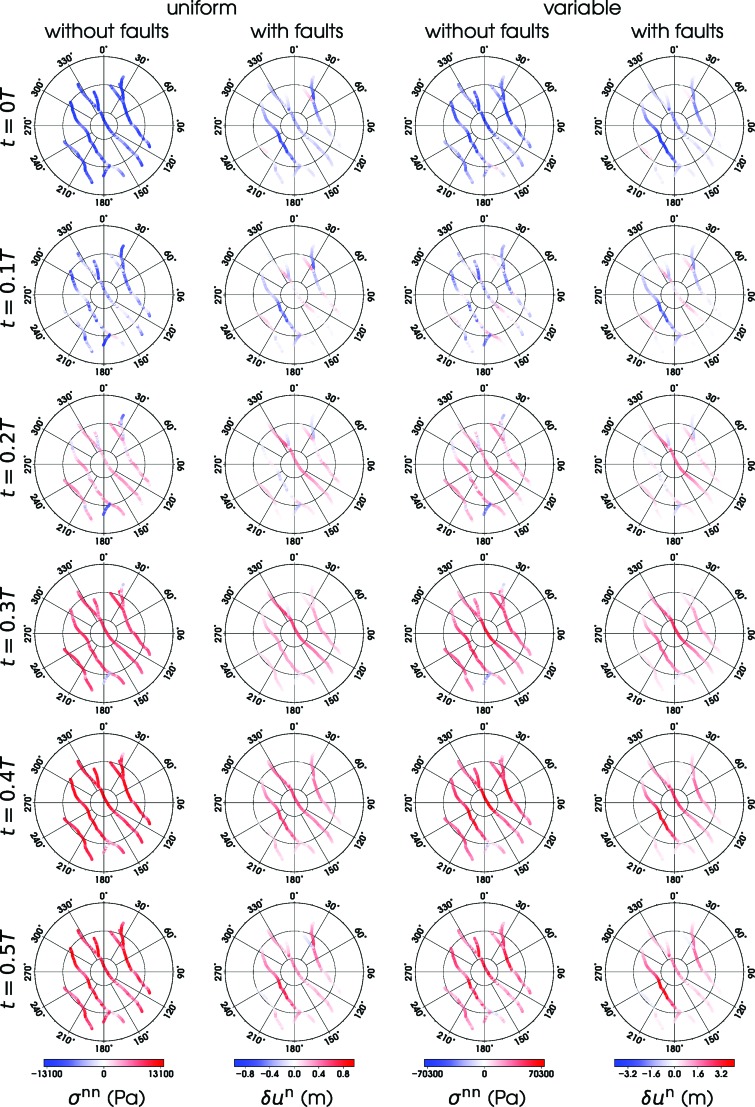
Opening rate of the faults in SPT computed in different orbital phases for the models considered in this study. The four columns correspond to models, from left to right, M^*U*^, M^*UF*^, M^*V*^, and M^*VF*^. The opening rate is expressed in terms of the normal traction vector for models without faults (M^*U*^ and M^*V*^) and in terms of the jump in the normal displacement for models with faults (M^*UF*^ and M^*VF*^). Positive value (red color) means that the fault is open. The south polar region is plotted in stereographic projection.

The activity predicted for the four models considered in this study is presented in Fig. 4, with open and closed segments marked in red and blue, respectively. The results are shown only for the first half of the period (*t* = 0 - *T/*2). Due to the symmetry properties of the tidal potential, the activity during the second half of the period can be obtained from the one in Fig. 4 by flipping the sign (for example, the activity pattern at time *t* = 0.6*T* can be obtained from that at *t* = 0.1*T* if the blue is replaced by red, and vice versa).

For model M^*U*^ (first column in Fig. 4), the activities of individual faults are in phase and of similar amplitudes, except for short marginal segments that differ in orientation from the main faults. The activity pattern obtained for model M^*V*^ (third column in Fig. 4) is similar to that of M^*U*^, but the activity varies among and along the faults, and the maximum amplitudes are significantly larger than in model M^*U*^ (note different color scales). The picture changes quite dramatically when the faults are included (model M^*UF*^, second column in Fig. 4), as then both the timing and the amplitude of activity become heterogeneous. This trend is further enhanced by including the variations in ice shell thickness (M^*VF*^, last column in Fig. 4).

**FIG. 5.**
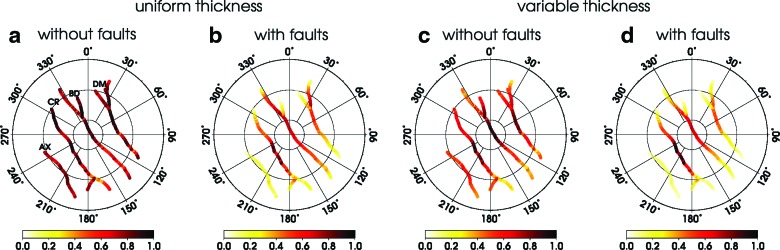
Predicted activity along the faults (0 least active, 1 most active) for models (**a**) M^*U*^, (**b**) M^*UF*^, (**c**) M^*V*^, and (**d**) M^*VF*^. The labels AX, CR, BD, and DM correspond to Alexandria sulcus, Cairo sulcus, Baghdad sulcus, and Damascus sulcus, respectively.

In the following, we assume that the local (time-dependent) activity at a point on the fault is proportional to the amplitude of the tensile normal traction component for models without faults or to the jump in normal displacement for models with faults. We then define the predicted activity for each point on the fault as the time average of the local activity over the tidal period, normalized to the maximum over all faults. The spatial variations in the predicted activity obtained in this way are shown in Fig. 5.

The activity predicted for model M^*U*^ (Fig. 5a) does not vary much along the fault lines, and it is approximately the same for all faults. In model M^*V*^ (Fig. 5c), the largest activity is concentrated in the central parts of the faults, and the activity decreases in peripheral regions where the ice shell thickness increases. The activity predicted for models with faults (Fig. 5b, 5d) strongly depends on the fault length and position, and it is significantly smaller for the outboard faults (Alexandria and Damascus) than for the inboard ones (Baghdad and Cairo).

The plume activity observed on Enceladus has been interpreted in two different ways. Porco *et al.* (2014) identified about 100 individual jets and showed that the eruptive activity is spatially heterogeneous and varies from fault to fault; they also suggested the possibility of interjet eruptions of sheets of material along broad regions of the fractures. The spatial heterogeneity of the eruptive activity predicted in our study is in agreement with the models including faults (Fig. 5b, 5d). Consistently, with Porco *et al.* (2014), these models also predict that Alexandria sulcus is the least active, although they do not reproduce the dominant role of Damascus sulcus. An alternative view was presented by Spitale *et al.* (2015), who argued that there were very few jets and the vast majority of the eruptive activity took the form of broad, curtainlike eruptions, with the activity decreasing toward the fault tips (Fig. 4f in Spitale *et al.,* 2015). From the perspective of our models, this concept only appears to be compatible with a scenario without faults (M^*V*^, Fig. 5c). Consequently, the recent arguments by Porco *et al.* (2015) in favor of the former scenario seem to support the important role of faults in the deformation of SPT.

**FIG. 6.**
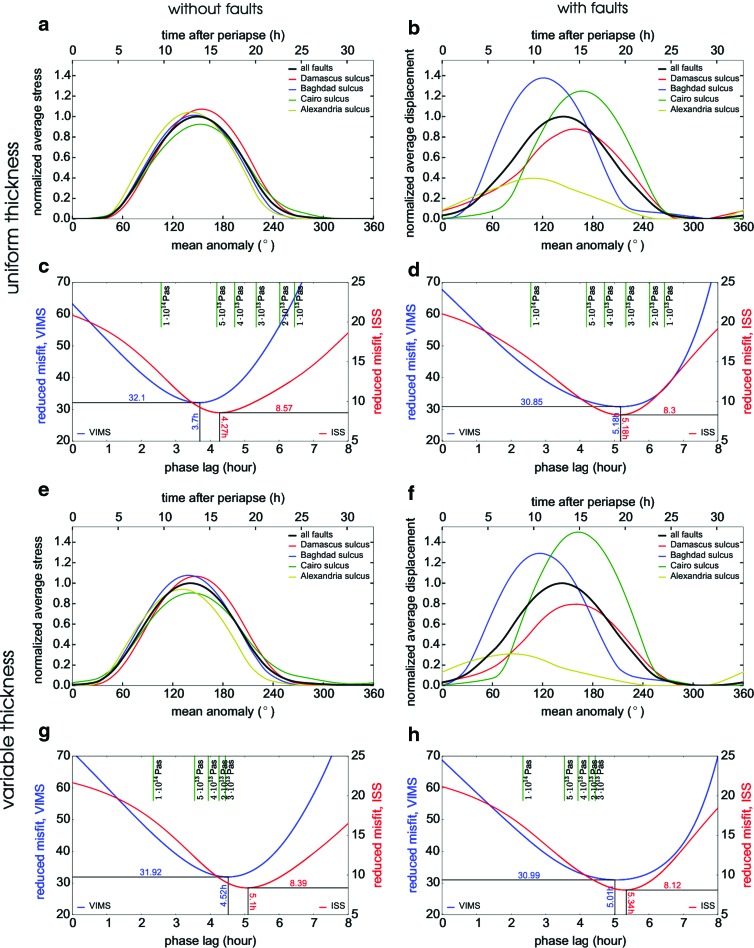
Activity of individual faults as a function of time predicted for models M^*U*^ (**a**), M^*UF*^ (**b**), M^*V*^ (**e**), and M^*VF*^ (**f**). The faults are distinguished in color. The black line shows the average activity over all faults. The misfit between the predicted average activity and the observed activity (blue VIMS Hedman *et al.* [2013], red ISS Nimmo *et al.* [2014]) plotted as a function of phase lag is shown for the same models in panels **c**, **d**, **g**, and **h**, respectively. The green vertical lines in the upper part of each panel mark the phase lags predicted for different values of viscosity using results by Nimmo *et al.* (2014).

Next, we compare the predicted and observed timing of the plume. For each fault, we define the predicted instantaneous activity as the spatial average of the local activity along the fault at a given time. The instantaneous activity predicted for the individual faults is shown as a function of time in Fig. 6a–6b, 6e–6f. The curves are normalized with respect to the maximum of the instantaneous activity computed for all faults (plotted in black). The inspection of Fig. 6a and 6e shows that, for the models without faults, the activities of the faults are all in phase, independently of whether variations in ice shell thickness are included or not. The inclusion of the faults (Fig. 6b, 6f) results in temporal decorrelation of the fault activities. The activity maxima predicted for Alexandria and Baghdad sulci are advanced by several hours with respect to Damascus and Cairo sulci, and the curves significantly differ in amplitudes. Although the times in which the individual faults reach the maximum activity differ by more than 6 h, the average activity over all faults remains almost the same as in the models without faults (compare black lines in Fig. 6a–6b, 6e–6f).

The predicted average activities are advanced by approximately 4.5–5.5 h with respect to the VIMS and ISS observations. Since the tidal response is computed for an elastic body, the phase lag might be attributed to the neglected effects of viscoelasticity. To test this hypothesis, we first use the method of Nimmo *et al.* (2014) to identify the lag best fitting the VIMS and ISS data, and attempt to interpret it in terms of viscosity of a Maxwell viscoelastic body. The reduced misfit (the misfit divided by the number of the degrees of freedom) as a function of the phase lag is shown in Fig. 6c–6d, 6g–6h. The values of misfit between the predicted and observed data are slightly lower for models with faults, although the difference is not significant. The best-fitting phase lag is smaller for models without faults than for models with faults, and it attains its absolute minimum for model M^*U*^ (*cf.* Nimmo *et al.,* 2014; Běhounková *et al.,* 2015). In the models without faults (Fig. 6c, 6g), the best-fitting phase lag is by 0.5 h smaller for the VIMS data than for the ISS data. This discrepancy, already noticed by Běhounková *et al.* (2015), is reduced (<0.25 h) when the faults are taken into account (Fig. 6d, 6h).

If we assume that the phase lag in the observed timing originates in the delayed response of a Maxwell viscoelastic body, the value of viscosity required to explain the best-fitting lag can be inferred based on the work of Nimmo *et al.* (2014, Fig. 11a). For models with constant ice shell thickness, the required viscosity is quite low, between 4 · 10^13^ and 1 · 10^14^ Pa s. Moreover, for models with variable ice shell thickness, even the largest possible phase lag (obtained for viscosity ∼3 · 10^13^ Pa s) cannot explain the discrepancy between the model and the observations. Note that this discrepancy would be even more pronounced in the case of out-of-phase libration. This suggests that either the ice shell thickness in the SPT is larger and/or our interpretation of fault activity in terms of traction/displacement along the faults is oversimplified and some important physical mechanism is possibly still missing in the description of the fault activation. Such a mechanism could be, for example, the hydrology of the fault zone (Kite and Rubin, 2016) and/or the interaction of faults closing/opening with ocean pressurization during the tidal cycle.

## 6. Discussion and Conclusions

In this article, we have investigated the impact of faults and ice-shell thickness variations on the spatial and temporal distribution of the tidal deformation and heating on Enceladus and their implications for the timing of the plume activity in the SPT. We performed numerical simulations of the tidal deformation for four structurally different models of Enceladus' ice shell (uniform/variable thickness, with/without faults), assuming purely elastic response of the ice. The model of ice shell thickness variations (Čadek *et al.,* 2016) considered in this study has recently been derived from gravity, topography, and libration data. The ice shell thickness in this model varies from a few kilometers in the SPT to more than 30 km in the equatorial regions. The faults that mimic Enceladus' tiger stripes are modeled as zones of reduced elastic moduli that pass vertically through the whole thickness of the shell (Souček *et al.,* 2016). This approximation corresponds to the idealized case where the faults are replaced by open slots of finite width (∼1 km) that do not transmit stress and do not dissipate energy. Consequently, our results give upper estimates of the impact of the faults on the tidal deformation of Enceladus, but they cannot be used to estimate the heat directly emanating from the faults.

In models without faults, the thinning/thickening of the ice shell leads to the increase/decrease in both the stress and the displacement. We find that, in first approximation, the stress is inversely proportional to the local thickness of the ice shell, which leads to a significant increase in stress amplitudes around the south pole. The effect of ice shell thickness variations on the displacement is less pronounced, and a further study that includes realistic (viscoelastic) rheology will be needed to quantify this effect in detail. The faults on the south pole have a negligible impact on the state of stress at the global scale, but their effect on the deformation and stress pattern in the SPT is profound. The inclusion of the faults in the model promotes an overall enhancement of deformation in the SPT, with the maxima of displacement being located in between the faults. A remarkable synergistic effect is obtained when the faults are combined with the polar ice shell thinning, leading to an increase in deformation in the SPT by an order of magnitude compared to the uniform thickness model without faults. The stress and strain concentrate at the tips of the faults and decrease in zones in between the faults.

Using the correspondence principle, we can relate the tidal stress in an elastic shell with the tidal heating for the Maxwell rheology. It can be shown (Souček *et al.,* 2016) that under certain conditions the heating is proportional to the time average of the square of the deviatoric stress; consequently, both heating and stress share similar spatial characteristics—the tidal heating strongly increases in regions with reduced ice shell thickness and is further enhanced in the vicinity of the fault tips. The maxima of tidal heating are located at the boundary between the tectonic units csp and cl_3_ (Crow-Willard and Pappalardo, 2015), where detailed analysis of the microwave radiometry observations reveals heat flux anomalies reaching 1–2 W m^−2^ (Le Gall *et al.,* 2017). Our model predicts the tidal heating maximum of 1.3 W m^−2^ for the value of viscosity 10^14^ Pa s, which is in surprisingly good agreement with the observations.

We have used the estimated heat production together with a simple estimate of conductive heat losses to investigate the stability of the shell in terms of local melting/freezing conditions at its base. Considering the extreme case where the heat sources in Enceladus' ocean and core are neglected, we find that viscosity lower than 5 · 10^13^ Pa s is needed in order to prevent the crystallization beneath the SPT for models without faults, and only a slightly higher value (10^14^ Pa s) is needed for models with faults. To prevent global crystallization, viscosity of the order of 10^13^ Pa s would be necessary.

Our results suggest that the faults have a large impact on prediction of the jet activity and increase its spatial and temporal heterogeneity, while the variations in ice shell thickness mainly influence the activity amplitude. The spatial heterogeneity is manifested by higher activity of the inboard faults (Baghdad and Cairo) compared to the outboard ones (Alexandria and Damascus) and by an increase of activity toward the center of the faults. Due to interaction between the faults, the activity maxima of the individual faults are shifted from each other by several hours. If the properties of the ejected particles differ from fault to fault (see, *e.g.,* Dhingra *et al.,* 2017), the heterogeneity of fault activity might lead to a detectable temporal variation of the plume composition. The ∼5 h phase lag of the observed jet activity with respect to the tidal force can be explained by using a Maxwell viscoelastic model if the average value of viscosity is lower than 5 · 10^13^ Pa s (*cf.* Nimmo *et al.,* 2014).

Remarkably, the values of viscosity needed to explain the time lag are similar to those needed to reach the melting regime in the SPT. Such average values of viscosity are, however, unrealistically low, at least compared to the available estimates of ice viscosity based on laboratory experiments and mineral physics (*e.g.,* Goldsby and Kohlstedt, 2001; Duval, 2013), and they can only be obtained at relatively high temperatures for small grain size and/or large stress, or if the ice is partially molten (De La Chapelle *et al.,* 1999; Tobie *et al.,* 2003). These conditions correspond to a vigorously convecting ice shell—a scenario that is unlikely due to the small thickness of the shell. The prevailing heat transport mechanism in the ice shell is likely to be the heat conduction (Barr and McKinnon, 2007), establishing a predominantly vertical temperature gradient and resulting in very high viscosity in most of the shell. The values of the global tidal heating in the ice shell presented here should therefore be regarded as upper limits, probably exceeding the actual value many times. If the average viscosity of the ice shell is higher than 5 · 10^13^ Pa s, which is highly probable, then additional heat sources in the deep interior are needed to maintain the subsurface ocean in global thermal equilibrium. The nature of possible heat sources in the deep interior has been discussed in a number of papers (Tyler, 2009; Roberts, 2015), and their existence has been indirectly confirmed by recent nanosilica observations (Hsu *et al.,* 2015). The amount of the additional heat power required to keep the ocean in a steady state increases with the viscosity of the ice shell and is approximately 10 GW already for a value of 10^14^ Pa s. The heat loss of Enceladus controlled by the conduction in the ice shell is thus compensated by interior heat sources of the order of magnitude of 10 GW. This estimate is consistent with the present-day prediction of the equilibrium heat production based on the estimate of the Q-factor in Saturn (Lainey *et al.,* 2012, 2017) and on the tidal evolution models (Meyer and Wisdom, 2007; Fuller *et al.,* 2016).

The prediction of the overall jet activity is a puzzling problem. For an ice shell that is 25 km thick on average, a satisfactory fit to the brightness data is achieved only for unrealistically low values of viscosity, independently of whether the model includes faults or not. The worst prediction is obtained for the models with variable thickness, which cannot explain the phase lag larger than 4.5 h. This indicates that either the ice shell thickness in the SPT in the model of Čadek *et al.* (2016) is underestimated (*cf.* van Hoolst *et al.,* 2016) or the applied parameterization of inelasticity, based on the Maxwell model with a constant viscosity, is oversimplified (*cf.* also Běhounková *et al.,* 2015). The delay of the jet activity with respect to the tidal loading may also have other reasons, such as a different or more complex geometry of the faults (*cf.* geometry of active faults in Porco *et al.,* 2006, 2014), friction and other dissipative processes at the faults, hydrological processes in the fault zones such as dynamic water-filling of the slots (Kite and Rubin, 2016), porous flow constrained by permeability of the ice shell (De La Chapelle *et al.,* 1999), and/or dynamic processes in the ocean. It should be noted that our knowledge of the inelastic deformation of ice on the scale of hours and hundreds of kilometers is incomplete, and further research will be needed to confirm whether the material models of the Maxwell type (*e.g.,* Castillo-Rogez *et al.,* 2011; Efroimsky, 2012; McCarthy and Castillo-Rogez, 2013) yield a reasonable approximation of the dissipative processes in icy moons.

We addressed the influence of faults and uneven ice shell thickness on tidal deformation, tidal heating, and plume activity on Enceladus. We found a remarkable increase in stress, deformation, and tidal heating in the SPT if realistic ice shell thickness and faults are included. The presence of faults results in large spatial and temporal heterogeneity of the predicted plume activity with a possible impact on time variability in the plume composition. The model presented in this study may help to better understand the nature of the faults and to improve the activity prediction. Such information may be valuable for future missions searching for evidence of life on Enceladus.

## Abbreviations Used

ISS: Imaging Science Subsystem
SPT: south polar terrain
VIMS: Visible and Infrared Mapping Spectrometer
